# Convergent, Parallel and Correlated Evolution of Trophic Morphologies in the Subfamily Schizothoracinae from the Qinghai-Tibetan Plateau

**DOI:** 10.1371/journal.pone.0034070

**Published:** 2012-03-28

**Authors:** Delin Qi, Yan Chao, Songchang Guo, Lanying Zhao, Taiping Li, Fulei Wei, Xinquan Zhao

**Affiliations:** 1 Animal Science Department of Agriculture and Animal Husbandry College, Qinghai University, Xining, Qinghai, China; 2 Key Laboratory of Qinghai-Tibetan Plateau Biological Evolution and Adaptation, Northwest Plateau Institute of Biology, The Chinese Academy of Sciences, Xining, Qinghai, China; Biodiversity Insitute of Ontario - University of Guelph, Canada

## Abstract

Schizothoracine fishes distributed in the water system of the Qinghai-Tibetan plateau (QTP) and adjacent areas are characterized by being highly adaptive to the cold and hypoxic environment of the plateau, as well as by a high degree of diversity in trophic morphology due to resource polymorphisms. Although convergent and parallel evolution are prevalent in the organisms of the QTP, it remains unknown whether similar evolutionary patterns have occurred in the schizothoracine fishes. Here, we constructed for the first time a tentative molecular phylogeny of the schizothoracine fishes based on the complete sequences of the cytochrome *b* gene. We employed this molecular phylogenetic framework to examine the evolution of trophic morphologies. We used Pagel's maximum likelihood method to estimate the evolutionary associations of trophic morphologies and food resource use. Our results showed that the molecular and published morphological phylogenies of Schizothoracinae are partially incongruent with respect to some intergeneric relationships. The phylogenetic results revealed that four character states of five trophic morphologies and of food resource use evolved at least twice during the diversification of the subfamily. State transitions are the result of evolutionary patterns including either convergence or parallelism or both. Furthermore, our analyses indicate that some characters of trophic morphologies in the Schizothoracinae have undergone correlated evolution, which are somewhat correlated with different food resource uses. Collectively, our results reveal new examples of convergent and parallel evolution in the organisms of the QTP. The adaptation to different trophic niches through the modification of trophic morphologies and feeding behaviour as found in the schizothoracine fishes may account for the formation and maintenance of the high degree of diversity and radiations in fish communities endemic to QTP.

## Introduction

The Qinghai-Tibetan plateau (QTP), which is renowned as being “the roof of the world”, is the world's largest high-elevation ecosystem, occupying nearly 2.5 million km^2^, with an average elevation of more than 4,000 m above sea level [1]. This region, along with southeast China and the Himalayan biodiversity hotspot, has been designated as one of the world's 34 most important centres of biodiversity because of its high species richness and abundance of endemic species [2], [3]. The mechanisms leading to these high species richness and the evolutionary patterns producing morphological diversity are not well understood.

Convergence and parallelism, two important trajectories in morphological evolution, have been the focus of evolutionary research, and could be distinguished by examining the phenotypic trajectories along a molecular phylogeny [4], [5]. Convergent evolution is the process by which lineages with different ancestral morphologies can independently evolve in different trajectories towards the same adaptive phenotype. Alternatively, lineages may begin with the same ancestral morphology and may evolve in the same direction towards a new, but similar adaptive phenotype. Convergence and parallelism have been amply discussed in evolutionary patterns including morphological, ecological, and behavioural traits in most major fish lineages [6]–[11]. Previous studies revealed that convergent and parallel evolution to be prevalent in the plants distributed in the QTP [12]–[15]. Similar cases were also reported for fishes. The species of *Sinocyclocheilus* occurs in the eastern QTP and adjacent areas. It exhibits degeneration of the eyes and pigments, and a well-developed projection of frontal and parietal bones that adapt to cave environments. Through molecular analyses these have been demonstrated to having originated multiple times from different lineages during the evolutionary history of this genus [8]. In contrast, it remains unknown whether similar evolutionary patterns have occurred in the schizothoracine fishes distributed in the QTP, due in part to the difficulty in obtaining specimens.

Here, we used the schizothoracine fishes as a model group to study convergence and parallelism. This group, recognized as a subfamily of the Cyprinidae, comprises about 11–12 genera and ca. 100 species [16]. In China, more than 70 recognized species account for nearly 80% of the world's schizothoracine species, and are mainly distributed in cold tributaries and lakes of the QTP and adjacent areas at 2000 m above sea level [17], [18]. The schizothoracine fishes confined to regions at either high altitudes or high latitudes have evolved a series of both morphological and physiological traits to adapt to the cold and hypoxic environment, and play significant roles in the trophic web of QTP freshwater communities [17]. The most striking feature is that the schizothoracine fishes exhibit a high degree of diversity in trophic morphologies to meet the demand for dealing with trophic polymorphisms (or resource polymorphisms) [17]. Several studies have discussed the phylogenetic relationships of the schizothoracine fishes based on molecular data, but these focused only on several genera instead of analysing all recognized genera of the schizothoracine fishes [19]–[22]. Therefore, the complete phylogenetic relationships within this subfamily including all recognized genera remain poorly understood.

In this study, we reconstructed for the first time a tentative molecular phylogeny based on the complete sequences of cytochrome *b* gene of the schizothoracine fishes including all recognized genera occurring in the QTP. We then employed this molecular phylogenetic framework to examine the evolution of trophic morphologies in the schizothoracine fishes, and used Pagel's maximum likelihood (ML) method [23] to estimate the evolutionary associations of trophic morphologies and food resource use. The objective of our study was to test (1) whether the molecular phylogeny is consistent with that based on morphological characters; (2) whether there are repeated evolutionary transitions in states of trophic morphologies; (3) when a character state has multiple origins, whether these lineages are the result of convergent evolution or parallel evolution and finally (4) whether there are evolutionary associations among trophic characters and food resource use.

## Results

### Molecular phylogeny

**Figure 1:**
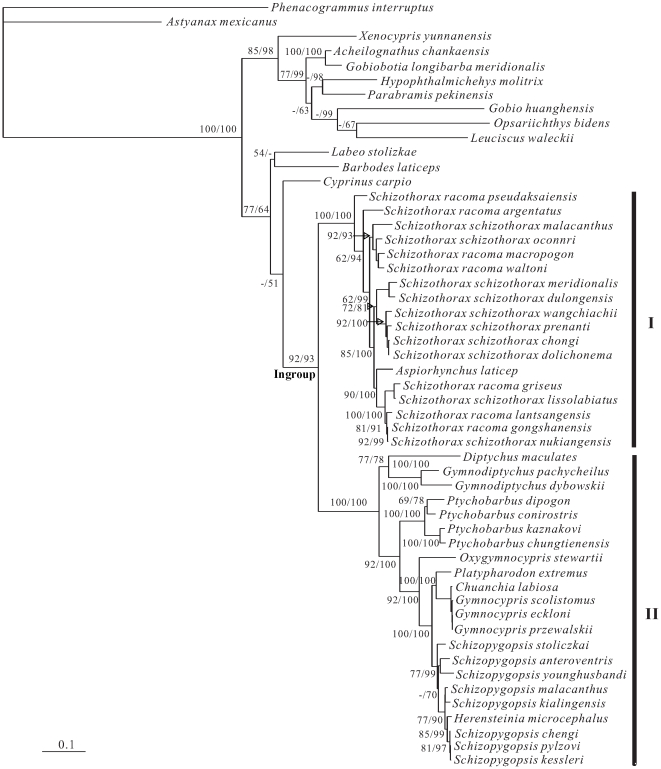
ML phylogenetic tree inferred from the complete cytochrome *b* gene sequences. The numbers on the branches correspond to bootstrap support (BP) for the ML tree and posterior probabilities (PP, shown as percentages) for the BI tree.

The best-fit model of molecular evolution obtained from ModelTest 3.06 based on the likelihood ratio tests was the GTR+I+G model. Settings for this model were as follows: Base = (0.3059 0.3405 0.0921), Nst = 6, Rmat = (0.6799 19.8065 0.7380 1.2627 9.1259), Rates = gamma, Shape = 0.8461, and Pinvar = 0.4785. Parameters obtained from this analysis were used for the construction of maximum likelihood (ML) and Bayesian inference (BI) phylogenies. Both ML and BI yielded similar trees with very similar branching patterns (Figure 1). The trees showed that the schizothoracine fishes are a well-supported monophyletic group (branch support [BP] = 92% in ML, and posterior probability [PP] = 93% in BI). Within this group, two major clades (I and II) were identified. Clade I contained the genus *Schizothorax* and the monotypic genus *Aspiorhynchus* with high statistical support (BP = 100% in ML, and PP = 100% in BI). Clade II contained the remaining genera (including the monotypic genera *Diptychus*, *Oxygymnocypris*, *Chuanchia*, *Platypharodon* and *Herensteinia*, and the genera *Ptychobarbus*, *Gymnodiptychus*, *Gymnocypris* and *Schizopygopsis*) also with high statistical support (BP = 100% in ML, and PP = 100% in BI).

### Evolution of trophic morphologies

#### Lower jaw morphology

**Figure 2:**
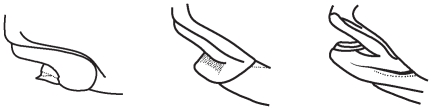
Three morphotypes of the lower jaw morphology found in the schizothoracine fishes. (A) With sharp outer horny sheath on the lower jaw. (B) With blunt outer horny sheath or inner horny membrane on the lower jaw. (C) Without horny sheath on the lower jaw.

**Figure 3:**
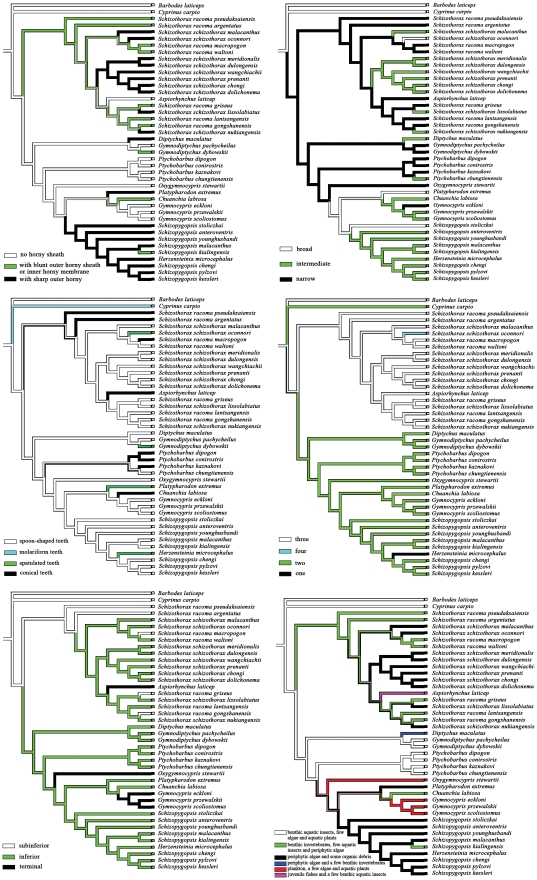
Evolution of the trophic morphologies in the schizothoracine fish. (A) Lower jaw morphology. (B) Shape of the pharyngeal bones. (C) Shape of the pharyngeal teeth. (D) Number of pharyngeal teeth rows. (E) Mouth position. (F) Food resource use. Character optimizations analyzed using maximum parsimony method in Mesquite.

The lower jaw morphology is strongly related to the food types. In general, for schizothoracine fishes, there are three morphotypes including no horny sheath, with blunt outer horny sheath or inner horny membrane and with sharp outer horny sheath on the lower jaw, and the definitions of the lower jaw morphology were adopted from Wu and Wu [17] (Figure 2). When the states of the lower jaw morphology for each species were mapped on the phylogenetic tree, the ancestral condition of this character for the whole clade could not be inferred (Figure 3A). The ancestral condition for the rest of Schizothoracinae was equivocal, due to the split into two large clades, each with a different ancestral condition: one possessed a blunt outer horny sheath or inner horny membrane (clade I), and the other exhibited no horny sheath (clade II). Within clade I, the lower jaw morphology showed six state transitions: five origins of sharp outer horny sheath (in the subspecies *schizothorax* of the genus *Schizothorax*) and one origins of no horny sheath (in the monotypic genus *Aspiorhynchus*). In the second clade (clade II), a sharp outer horny sheath evolved three times, in *Diptychus* (a monotypic genus), *Platypharodon* (a monotypic genus) and in *Schizopygopsis*. A blunt outer horny sheath or an inner horny membrane also evolved three times, in *Gymnodiptychus dybowskii*, *Chuanchia labiosa* (species of the monotypic genus *Chuanchia*) and in *Schizopygopsis kialingensis*.

#### Shape of pharyngeal bones

For the shape of pharyngeal bones, the ancestral state of Schizothoracinae, as inferred from the MP reconstruction, was resolved as narrow type (Figure 3B). From this ancestral condition, the intermediate pharyngeal bones evolved eight times and the broad pharyngeal bones evolved three times in the entire clade. One reversal to the ancestral state (narrow type) could also be noted in *Gymnocypris*.

#### Shape of pharyngeal teeth

The definitions for each tooth shape are taken from Chu [24]: (i) spoon-shaped teeth are conical teeth with a concave surface, a pointed tip and a hook; (ii) spatulated teeth are teeth which are compressed but with the apical regions swollen, closely aggregated and fitted together. The grinding surfaces are truncated and form together a common, round chewing area; (iii) conical teeth are simple teeth with a rounded tip. *Cyprinus carpio* has molariform teeth that are crushing teeth resembling “elephant's teeth” [24]. The ancestral condition of pharyngeal teeth shape for the whole clade could not be inferred, while MP estimations of the ancestral pharyngeal teeth shape suggest this character had a minimum of 11 state transitions during evolution (Figure 3C). These transitions included four independent origins of spatulated teeth from spoon-shaped teeth and seven origins of conical teeth from spoon-shaped teeth.

#### The number of pharyngeal teeth rows

The schizothoracine fishes may have one, two, three or four rows of pharyngeal teeth. The parsimony reconstructions according to tree topology unambiguously inferred three rows of pharyngeal teeth as the ancestral state for clade I and two rows of pharyngeal teeth as the ancestral state for clade II, although the ancestral condition of this character for the whole clade could not be resolved (Figure 3D). From an ancestral condition of three and four rows of pharyngeal teeth, each evolved once in *Schizothorax* in clade I. Within clade II, one row of pharyngeal teeth evolved once in *Herensteinia*.

#### Mouth position

Concerning mouth position, MP estimations of the ancestral states identified a minimum of seven state transitions: one from subinferior to inferior, one from inferior to terminal, and three reversals to the ancestral state (subinferior mouth) in clade I, while two from inferior to terminal were observed in clade II (Figure 3E).

### Ancestral condition of food resource use

The ancestral condition of food resource use for the whole clade could not be inferred, while the two large clades had different ancestral conditions of food resource use: one (clade I) fed on benthic invertebrates, few aquatic insects and periphytic algae, and the other (clade II) was feeding on benthic aquatic insects, few algae and aquatic plants (Figure 3F).

### Convergent versus parallel evolutionary events

Based on phylogenetically-based definitions of convergence and parallelism [4], [5], we examined the number of convergent versus parallel evolutionary events during the trophic characters evolution of schizothoracine fishes. For the lower jaw morphology, all five origins of a sharp outer horny sheath in clade I were parallel trajectories arising from an ancestor with a blunt outer horny sheath or an inner horny membrane on the lower jaw. Likewise, three origins of a sharp outer horny sheath and three origins of a blunt outer horny sheath or inner horny membrane in clade II were results of parallel evolution. The sharp outer horny sheaths of clade I and clade II were convergent and derived from an ancestor with a blunt outer horny sheath or an inner horny membrane on the lower jaw and an ancestor with no horny sheath on the lower jaw. Both the intermediate and broad pharyngeal bones arose in parallel from an ancestor with narrow pharyngeal bones. For the shape of the pharyngeal teeth, all state transitions were parallel trajectories arising from an ancestor with spoon-shaped teeth. The terminal mouth arose in two independent lineages (clade I and II) along both parallel and convergent trajectories, while three reversals to the ancestral state (subinferior mouth) were due to parallel evolution. Furthermore, the four rows and one row of pharyngeal teeth were both unique in the schizothoracine fishes and originated from a three rows ancestor and a two rows ancestor, respectively.

The likelihood ratio test comparing the two models for character evolution did not reject the null hypothesis for all characters of trophic morphology because both models never differed significantly from each other at a liberal α value (Table 1). This suggested that the one-parameter model, in which forward = backward, was favoured over the two-parameter asymmetrical model (forward/backward model). The test was significant at 99% confidence level for food resource use.

**Table 1:** Likelihood ratios test comparing two models for character evolution of trophic morphologies and food resource use (diet).

Hypothesis	LJM	PB	PTS	PTR	MP	Diet
ln(likelihood H_0_)	−38.82±0.89	−38.60±0.74	−34.44±0.69	−15.65±0.49	−34.10±1.17	−52.95±1.10
ln(likelihood H_1_)	−38.73±0.88	−37.43±0.56	−33.73±0.46	−15.51±0.48	−33.84±1.12	−49.57±1.06
LR test	0.18	2.34	1.42	0.28	0.52	6.76
*P*	0.67	0.13	0.23	0.56	0.47	0.009

Mean values ± standard deviations are shown. H_0_ is the one-parameter model, in which forward = backward. Morphological characters and character coding used in this study: LJM, lower jaw morphology; PB, shape of the pharyngeal bones; PTS, shape of the pharyngeal teeth; PTR, pharyngeal teeth rows; MP, mouth position.

### Evolutionary associations between trophic morphologies and food resource use

The results from the Pagel's ML analysis showed that the results of all pair-wise comparisons between lower jaw morphology and shape of pharyngeal bones, number of pharyngeal teeth rows and food resource use, shape of pharyngeal bones and number of pharyngeal teeth rows, number of pharyngeal teeth rows and mouth position and food resource were significantly or very significantly associated (Bayes factor >5; see Table 2). For example, there were three character states of lower jaw morphology on the tree seen in Figure 3A. These included no horny sheath, with blunt outer horny sheath or inner horny membrane and with sharp outer horny sheath, which corresponded to three combinations of food resource use on the tree in Figure 3F: feeding on benthic aquatic insects, few algae and aquatic plants+juvenile freshwater fishes and a few benthic aquatic insects+plankton, few algae and aquatic plants; benthic invertebrates, few aquatic insects and periphytic algae; periphytic algae and some organic debris+periphytic algae and a few benthic invertebrates. These significant evolutionary associations indicated that all these characters were either functionally correlated during the evolutionary process or linked to the specific food resource use.

**Table 2:** Bayes factor (BF) calculated in searching for correlated evolution of trophic morphologies and food resource use (diet) in the Schizothoracinae.

	LJM	PB	PTS	PTR	MP	Diet
LJM	–					
PB	0.94	–				
PTS	6.30*	1.68	–			
PTR	13.22**	10.92**	1.6	–		
MP	3.84	0.06	2.64	5.24*	–	
Diet	13.68**	3.24	4.18	7.52*	2.58	–

Morphological characters and character coding are the same as in Table 1. BF>2, positive evidence;

* BF>5, strong correlation;

** BF>10, very strong evidence.

## Discussion

The subfamily Schizothoracinae is defined as a natural group highly adaptive to the extreme environment of the QTP [17], [25]. The present analysis based on molecular data shows that the schizothoracine fishes form a well-supported monophyletic group, In Wu's [25] phylogenetic analysis, all species of the Schizothoracinae clustered as three major clades including primitive, specialized and highly specialized clades. The primitive clade consists of *Schizothorax* and *Aspiorhynchus*, being characterized by having uroneuralia, three or four rows of pharyngeal teeth, less degeneration of scales and indistinct sexual dimorphism, and exhibited more traits similar to the out-group *Barbodes hexagonolepis*. The specialized clade includes *Ptychobarbus*, *Gymnodiptychus* and *Diptychus*, with one or two rows of pharyngeal teeth, absence of uroneuralia, significant and moderate degeneration of scales. The highly specialized clade is composed of the remaining five genera, *Gymnocypris*, *Oxygymncypris*, *Schizophygopsis*, *Chuanchia* and *Platypharodon*, being characterised by a total absence of barbels and scales, as well as a well-developed canalis preoperculomandibularis. However, our molecular analysis showed that this subfamily formed two major clades, which is not consistent with the previous morphological phylogenetic analysis. Morphological results of Wu and Wu [17] and Wu [25] revealed that species from *Schizothorax* clustered as two reciprocally monophyletic groups corresponding to the two distinct genera *Schizothorax* and *Racoma*, which clustered together and formed a sister group of the monotypic genus *Aspiorhynchus laticeps*. In the most recent classification, the genus *Racoma* was incorporated into the genus *Schizothorax*, but was divided into the two subgenera *Schizothorax* and *Racoma* [18]. However, our phylogenetic study clearly showed that the species from the two genera (or two subgenera) *Schizothorax* and *Racoma* were intermingled, and the monotypic genus *Aspiorhynchus* embedded within them. In fact, incongruence between morphological and molecular phylogenies has been recognized and debated ever since molecular techniques have been in use [26]. Similar phenomena have also been observed in some fishes and mammals [27]–[29]. *Schizothorax* is characterized by having a sharp outer horny sheath on the lower jaw, while *Racoma* possess a blunt outer horny sheath or an inner horny membrane. Based on the lower jaw morphology, Wu and Wu [17], Wu [25] and Chen and Cao [18] classified them into two distinct genera or subgenera. Both *Schizothorax* and *Racoma* are widely distributed in all drainages of the QTP except for the Qiadam Basin, Huangshui River and some isolated lakes. Some of the characters used for taxonomic assignments of this lineage, including the lower jaw morphology, arrangement of pharyngeal teeth and mouth positions, were suggested to result from adaptive evolution to the high degree of resource polymorphisms and habitat shifts [17], [25], [30]. Fluctuations of the ecogeographical environment occurred in the course of uplifting of the QTP and might have caused repeated loss and gain of some adaptive morphological traits during speciation within the plants and animals distributed in this region. Previous studies revealed that convergent and parallel evolution are common in plants and small animals distributed in the QTP [8], [12]–[15]. The molecular phylogeny presented here might indicate that the lower jaw morphology was phylogenetically constrained because of convergent and parallel evolution. In addition, Chu [24] first established the monotypic genus *Herzensteinia* according to the distinctive morphological characters. Based on morphological and osteological characters, Wu and Wu [17] and Wu [25] incorporated the genus *Herzensteinia* into the genus *Schizopygopsis* as a species named *Schizopygopsis microcephalus*. However, Chen and Cao [18] continued to consider the genus *Herzensteinia* valid. The results of our analyses show that *Herzensteinia microcephalus* is closely related to the genus *Schizophygopsis*, and that together they form a monophyletic group with strong bootstrap support. Therefore, further studies that would add new sequence data (including mitochondrial and nuclear gene sequences) and additional taxa (including all recognized species of Schizothoracinae) are required to test the overall phylogenetic relationships among the Schizothoracinae.

While patterns of convergence, parallelism and evolutionary association of trophic morphologies are well-documented in other fishes [6]–[11], it remains unknown whether such evolutionary patterns have occurred in the schizothoracine fishes. Our study represents a major contribution to understanding repeated patterns of evolution and correlated evolution of trophic morphologies in the Schizothoracinae. Character states of four of all five trophic morphologies and use of food resources evolved at least twice during the diversification of the subfamily. Our data support the hypothesis that species with identical trophic morphologies evolved independently and concurrently in different drainage systems. Interestingly, a sharp outer horny sheath is the most common character state of the lower jaw morphology, which evolved independently eight times through both convergent and parallel evolution. From the ancestral condition of narrow pharyngeal bones, the intermediate pharyngeal bones evolved eight times and became the most common character state of pharyngeal bones. The replicated evolution of morphologically divergent species pairs points to the presence of well-defined trophic niches that have facilitated ecological segregation, and to the adaptive value of the observed morphological associations. The morphological covariation of species from the subfamily Schizothoracinae (e.g. species with a sharp outer horny sheath on the lower jaw have intermediate pharyngeal bones, spoon-shaped teeth, as well as an inferior or subinferior mouth) is produced by similar selective pressures and functional constraints, enabling the multiple independent invasions of the same adaptive zone as suggested by Rüber and Adams [6].

Differences in trophic morphologies in closely related fishes or ecomorphs of the same species are often correlated with tradeoffs for resource use [31]–[33]. The distinct trophic morphologies found in cichlid fishes [33], [34] and Gobioninae [10] are correlated with differences in diet. It is interesting to note that the trophic morphologies of schizothoracine fishes in this study are somewhat correlated with the use of different food resources. The sharp outer horny sheath on the lower jaw, always accompanied by an inferior or subinferior mouth, spoon-shaped teeth, as well as intermediate pharyngeal bones, are found in scrapers, such as species from the genera *Schizopygopsis*, *Herzensteinia* and the subgenus *Schizothorax*. They live in fast flowing currents and feed primarily on periphytic algae (e.g. diatoms growing on solid substrate, such as stones), as well as on a small quantity of benthic invertebrates and organic debris, showing good adaptation to scraping function. Species without a horny sheath on the lower jaw always have a terminal mouth, as well as a well-developed selection apparatus consisting of gill rakers, gill arches and the palatal organ. These features are discovered in filter feeders, such as the species from the *Gymnocypris* and *Ptychobarbus* groups, which mainly live on plankton and benthic aquatic insects, including some aquatic plants and algae. They are well adapted to filtering function as strainers. The species (e.g. *Gymnocypris*) feeding primarily on plankton always have a terminal mouth and differ from those (e.g. *Ptychobarbus*) feeding primarily on benthic aquatic insects, which always have an inferior mouth. Species with a blunt outer horny sheath on the lower jaw, always accompanied by a terminal mouth, are benthic invertebrate feeders, such as the species from the subgenus *Racoma*, which mainly feed on benthic invertebrates and algae, thereby meeting the demand for a crushing and scraping function.

It is accepted that resource competition probably plays an important role in the evolution of diversity in many adaptive radiations. The adaptation to different trophic niches through the modification of trophic morphologies and feeding behaviour as found in the schizothoracine fishes may account for the formation and maintenance of the high degree of diversity and radiations in fish communities endemic to the QTP.

## Materials and Methods

### Ethics statement

All research involving animals in this study followed the guidelines of the regulations of experiments on animals, and has been approved by the Animal Care and Use Committee of the Qinghai University, China.

### Fish samples

**Figure 4:**
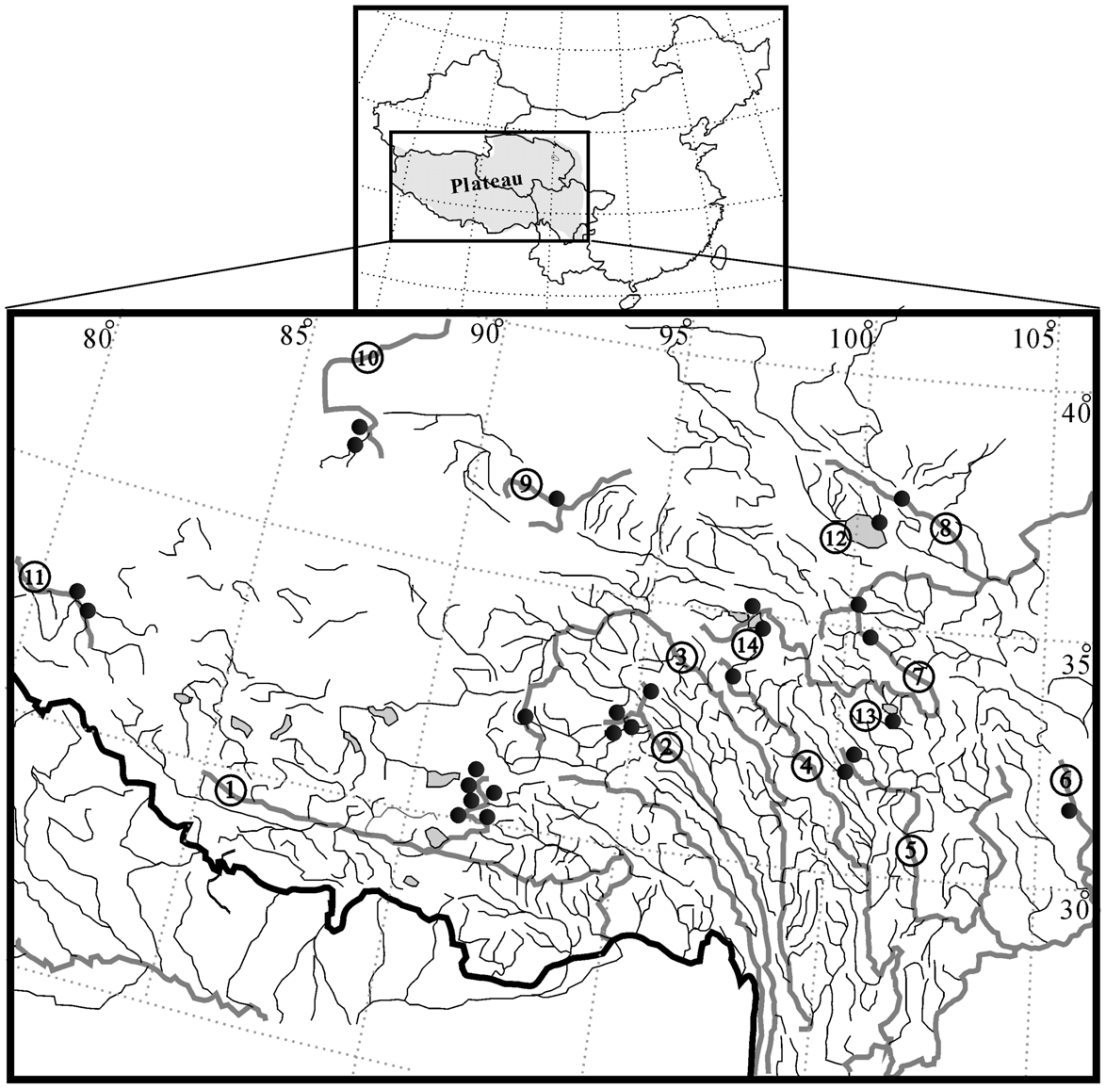
Sampling sites for schizothoracine fishes from the Qinghai–Tibetan Plateau. The numbers refer to the catchments listed in Table S1.

A total of 40 species, including representatives from all recognized genera of the Schizothoracinae, were examined (Figure 4). Of these, 13 species sequences were downloaded from GenBank. The others were collected as live specimens from their distributed regions during 2004–2011 using gill nets or cast nets. (See Table S1, published as supporting information on the PLoS One web site.) The habitats of in-group taxa covered the main distribution drainages of schizothoracine fishes, including outflow drainages (Yellow River, Yangtze River, Nujiang River, Huangshui River, Lancang River, Indus River and Irrawaddy River), inflow drainages (Tarim River, Yili River, Qiadam Basin) and an isolated lake (Qinghai Lake). All specimens were preserved in 95% ethanol, or stored at −70°C for laboratory analyses. Voucher specimens were deposited at the Fishery Environmental Monitoring Station of Qinghai Province, China. In order to test the monophyly of Schizothoracinae, 11 species representing 11 subfamilies of Cyprinidae, and two other species belonging to the Alestiidae and Characidae, were used as the out-group [35].

### DNA extraction, PCR amplification and sequencing

Total DNA was extracted from ethanol-fixed or frozen tissue by using proteinase K digestion followed the phenol/chloroform extraction procedure [36]. The complete sequence of the cytochrome *b* gene (1140 bp) was amplified using the universal primers L14724 (5′-GACTTGAAAAACCACCGTTG-3′) and H15915 (5′-CTCCGATCTCCGGATTACAAGAC-3′) [37]. PCR amplifications were performed in a total reactions volume of 30 µl, containing 1.0 U TaKaRa EX Taq (TaKaRa, Dalian, China), 1 µl of dNTP mix (2.5 mM each), 3.0 µl of 10×Taq buffer (TaKaRa, Dalian, China), 0.5 µl of each primer (10 mM), and 0.1 µg of total genomic DNA. Reactions were carried out for initial denaturation at 94°C for 4 min, followed by 35 cycles of denaturation at 94°C for 1 min, annealing at 50°C for 1 min and extension at 72°C for 1 min, with a final extension at 72°C for 5 min. PCR products were purified using a CASpure PCR Purification Kit following the manufacturer's protocol (Casarray, Shanghai, China). The sequencing reactions were carried out in a Biometra thermocycler using a DYEnamic Dye Terminator Cycle Sequencing Kit (Amersham Biosciences Corporation, USA) according to the manufacturer's protocol. Purified DNA fragments were directly sequenced using a MegaBACE 500 DNA Analysis System following manufacturer instruction. To ensure accuracy, strands were sequenced in both directions for each individual. Both DNA strands were checked for ambiguous base assignments.

### Morphological observations and food resource use

**Figure 5:**
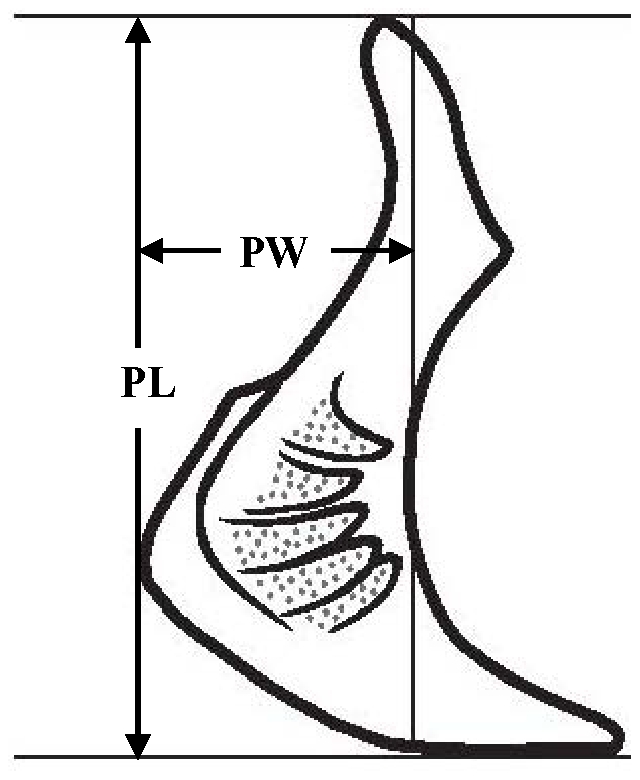
Pharyngeal bone showing the morphological traits and measurements in the present study. PL, length of pharyngeal bones; PW, width of pharyngeal bones.

To trace the evolutionary patterns and processes of trophic morphologies in the schizothoracine fishes, the lower jaw morphology, shape of the pharyngeal bones, shape of the pharyngeal teeth, number of pharyngeal teeth rows and the mouth position were observed. Morphological character data were retrieved from the literature [17], [25], except where more recent revisions were available [18]. Data not available in the literature were obtained by examining the voucher specimens for this study. (See Table S1, published as supporting information on the PLoS One web site.) The lower jaw morphology was graded as no horny sheath, with blunt outer horny sheath or inner horny membrane and with sharp outer horny sheath on the lower jaw, and the definitions of the lower jaw morphology were adopted from Wu and Wu [17] (Figure 2). The shape of the pharyngeal bones was identified as narrow, intermediate or short and broad type, with the ratio of the length of the pharyngeal bone to its width (PL/PW) ranging from 3.41 to 7.14, 2.41 to 3.4, and 1.17 to 2.0, respectively (Figure 5). For schizothoracine fishes, our investigations of individual tooth shape revealed three pharyngeal teeth in the main row. These spoon, spatula and conical dental morphotypes are consistent with those defined in previous studies [24], [38]. For the number of pharyngeal teeth rows, there were one, two, three or four rows of pharyngeal teeth. The mouth position was identified as subinferior, inferior or terminal.

Diversity of trophic morphologies was suggested as an adaptation to resource polymorphisms and feeding habits in Cypriniformes [9], [10]. In this study, we obtained the data of food resource use from the literature [17] and from analyzing stomach contents of wild specimens. For schizothoracine fishes, food resources could be divided into six types: (i) including benthic aquatic insects, few algae and aquatic plants; (ii) benthic invertebrates, few aquatic insects and periphytic algae; (iii) periphytic algae and some organic debris; (iv) periphytic algae and a few benthic invertebrates; (v) plankton, a few algae and aquatic plants; (vi) juvenile freshwater fishes and a few benthic aquatic insects.

### Data analysis

#### Molecular data analyses

Sequence alignment was conducted using CLUSTAL W [39] and checked manually. The new sequences have been deposited in the GenBank database under the accession numbers shown in Table S1.

The phylogenetic analyses were conducted using the maximum likelihood (ML) approach in PAUP* v4.0b10 [40], and Bayesian inference (BI) in MrBayes 3.0B4 [41]. The ML approach was used because it is one of the best performing phylogenetic methods, whereas BI performs exceptionally well in supporting correct grouping compared to traditional ML and maximum parsimony (MP) methods [42]. The best-fitting model of nucleotide evolution was selected by Modeltest version 3.06 [43]. For ML analysis, heuristic search parameters were simple addition sequence of taxa with TBR branch-swapping and 10 random sequence additions. Branch support (BP) for the ML tree was assessed using non-parametric bootstrapping [44] with 100 heuristic replicates with single random addition replicates.

For BI, four simultaneous Monte-Carlo Markov Chains of 5,000,000 steps were used, with a tree saved every 100 steps. Posterior probabilities (PP, shown as percentages) indicated branch support [45]. The first 5000 trees were discarded and 45,000 trees (whose log-likelihoods converged to stable values) were used to construct a 50% majority rule consensus tree with PP. The number of burn-in steps was determined by visual inspection of log-likelihood values.

### Morphological and food resource use analyses

Our aim was to reconstruct the evolutionary history of trophic morphologies (e.g. the lower jaw morphology, shape of the pharyngeal bones, shape of the pharyngeal teeth, number of pharyngeal teeth rows and mouth position), and to investigate the correlation between trophic characters and food resource use. Therefore, the trophic morphologies and food resource use were mapped onto the 50% majority rule consensus ML phylogenetic tree reconstructed with *Barbodes laticeps* and *Cyprinus carpio* as out-group (result not shown) because of their well-documented trophic morphologies and close relatives to schizothoracine fishes. Character evolution was then analyzed using the MP method in Mesquite version 2.74 for Windows [46], and all characters were treated as reversible and unordered.

A likelihood ratio test (LR test) was carried out with the program BayesMultiState by means of ML estimation techniques that use the Markov transition-rate model [23], [47], [48]. This was done by fitting a simpler model after the proper restriction of several parameters. One of the most typical simplifications is the nested model, in which only two parameters are calculated: the forward and backward rates. In this two-parameter asymmetrical model, a single forward parameter is calculated after restricting all forward parameters to be equal. Similarly, a single backward parameter is calculated once all the backward parameters have been constrained to be equal. The asymmetry in the model is derived from the fact that forward and backward rates are allowed to be different [48]–[50]. The LR test subtracts the smaller from the larger log-likelihood and distributes as a χ^2^ with degrees of freedom equal to the difference in the number of parameters between the two models. If the two models do not differ from each other at a liberal α value (*P*>0.10), then the one-parameter model, in which forward = backward, can be used to represent the data. Our H_0_ assumed that forward and backward transitions are equally likely.

### Estimating evolutionary associations

Pagel's ML method [23] was used to test evolutionary associations. As this method cannot deal with multi-state characters (>3), the coding method was employed as suggested by Zeng and Liu [10]. Pagel's ML method was performed with the software BayesTraits [51]. Statistical significance was accepted for *P*-values<0.05.

## Supporting Information

Table S1 Specimens, GenBank accession numbers and coding of morphological characters and diet composition for the analyzed samples.
(DOC)
