# Supplementary material for: Convergent, Parallel and Correlated Evolution of Trophic Morphologies in the Subfamily Schizothoracinae from the Qinghai-Tibetan Plateau
Source: PLoS One. 2012 Mar 28;7(3):e34070. doi: 10.1371/journal.pone.0034070 (PMC3314705; doi:10.1371/journal.pone.0034070)
Supplement: Table S1 — Specimens, GenBank accession numbers and coding of morphological characters and diet composition for the analyzed samples. (DOC) [file pone.0034070.s001.doc]

Table S1 Specimens, GenBank accession numbers and coding of morphological characters and diet composition for the analyzed samples

| Species | Collection location | Catchment (number in Fig. 4) | Drainage system | GenBank Accession No. | LJM | PB | SPT | PTR | MP | Diet |
| --- | --- | --- | --- | --- | --- | --- | --- | --- | --- | --- |
| **Alestiidae**  *Phenacogrammus interruptus* | GenBank |  |  | AY791434 |  |  |  |  |  |  |
| **Characidae**  *Astyanax mexicanus* | GenBank |  |  | AF045997 |  |  |  |  |  |  |
| **Danioninae**  *Opsariichthys bidens* | GenBank |  |  | AY646648 |  |  |  |  |  |  |
| **Leuciscinae**  *Leuciscus waleckii* | GenBank |  |  | AY026395 |  |  |  |  |  |  |
| **Labeoninae**  *Labeo stolizkae* | GenBank |  |  | GU086536 |  |  |  |  |  |  |
| **Xenocyprinae**  *Xenocypris yunnanensis* | GenBank |  |  | AF036208 |  |  |  |  |  |  |
| **Gobioninae**  *Gobio huanghensis* | Xinghai, Qinghai | Yellow River (7) | Yellow River | FJ904648* |  |  |  |  |  |  |
| **Hypophthalmichthyinae**  *Hypophthalmichthys molitrix* | GenBank |  |  | AF051866 |  |  |  |  |  |  |
| **Cultrinae**  *Parabramis pekinensis* | GenBank |  |  | AF051874 |  |  |  |  |  |  |
| **Gobiobotinae**  *Gobiobotia longibarba meridionalis* | GenBank |  |  | AF051874 |  |  |  |  |  |  |
| **Acheilognathinae**  *Acheilognathus chankaensis* | GenBank |  |  | AF375867 |  |  |  |  |  |  |
| **Barbinae**  *Barbodes laticeps* | GenBank |  |  | AY854739 | 0 | 0 | 0 | 0 | 0 | 0 |
| **Cyprininae**  *Cyprinus carpio* | GenBank |  |  | AY347280 | 0 | 0 | 1 | 2 | 0 | 0 |
| **Schizothoracinae**  *Schizothorax schizothorax o’connri* | Lhasa, Tibet | Lhasa River (1) | Yarlungzangbo River | JQ082339*  EF533719 | 2 | 0 | 2 | 1 | 1 | 2 |
| *Schizothorax schizothorax wangchiachii* | GenBank |  | Yangtze River | AY463521 | 2 | 1 | 0 | 0 | 1 | 2 |
| *Schizothorax schizothorax prenanti* | Banma, Qinghai | Dadu River (5) | Yangtze River | JQ082340* | 2 | 1 | 0 | 0 | 1 | 2 |
| *Schizothorax schizothorax lissolabiatus* | GenBank |  | Lancang River | EU158042 | 2 | 1 | 0 | 0 | 1 | 2 |
| *Schizothorax* *schizothorax chongi* | GenBank |  | Yangtze River | DQ126118 | 2 | 1 | 0 | 0 | 1 | 2 |
| *Schizothorax* *schizothorax dolichonema* | Zadoi, Qinghai | Zhaqu River (2) | Lancang River | JQ082344* | 2 | 1 | 0 | 0 | 1 | 2 |
| *Schizothorax* *schizothorax malacanthus* | GenBank |  | Irrawaddy River | AY954277 | 2 | 1 | 0 | 0 | 1 | 2 |
| *Schizothorax schizothorax* *meridionalis* | GenBank |  | Irrawaddy River | AY954285 | 2 | 1 | 0 | 0 | 1 | 2 |
| *Schizothorax* *schizothorax dulongensis* | GenBank |  | Irrawaddy River | AY954284 | 2 | 1 | 0 | 0 | 1 | 2 |
| *Schizothorax schizothorax nukiangensis* | GenBank |  | Nujiang River | DQ126125 | 2 | 1 | 0 | 0 | 1 | 2 |
| *Schizothorax racoma argentatus* | GenBank |  | Yili River | AF180861 | 1 | 2 | 3 | 0 | 0 | 1 |
| *Schizothorax racoma pseudaksaiensis* | GenBank |  | Yili River | AF180827 | 1 | 2 | 3 | 0 | 0 | 1 |
| *Schizothorax racoma macropogon* | Lhasa, Tibet | Lhasa River (1) | Yarlungzangbo River | JQ082341* | 1 | 2 | 3 | 0 | 0 | 1 |
| *Schizothorax racoma waltoni* | Lhasa, Tibet | Lhasa River (1) | Yarlungzangbo River | JQ082342* | 1 | 2 | 0 | 0 | 1 | 1 |
| *Schizothorax racoma lantsangensis* | Zadoi, Qinghai | Zhaqu River (2) | Lancang River | JQ082343* | 1 | 2 | 0 | 0 | 1 | 1 |
| *Schizothorax racoma gongshanensis* | GenBank |  | Nujiang River | AY954280 | 1 | 2 | 0 | 0 | 0 | 1 |
| *Schizothorax racoma griseus* | GenBank |  | Lancang River | AY954253 | 1 | 2 | 0 | 0 | 0 | 1 |
| *Aspiorhynchus laticep* | GenBank |  | Tarim River | GU814009 | 0 | 2 | 3 | 0 | 2 | 5 |
| *Ptychobarbus dipogon* | Lhasa, Tibet | Lhasa River (1) | Yarlungzangbo River | JQ082345* | 0 | 2 | 3 | 2 | 1 | 0 |
| *Ptychobarbus conirostris* | Gar, Tibet | Sênggê Zangbo River (11) | Indus River | JQ082346* | 0 | 2 | 3 | 2 | 1 | 0 |
| *Ptychobarbus kaznakovi* | Zadoi, Qinghai | Zhaqu River (2) | Lancang River | JQ082347* | 0 | 2 | 3 | 2 | 1 | 0 |
| *Ptychobarbus chungtienensis* | GenBank |  | Yangtze River | AY463508 | 0 | 1 | 0 | 2 | 1 | 0 |
| *Diptychus maculates* | Heshuo, Xinjiang | Longka River (10) | Tarim River | JQ082348* | 2 | 1 | 0 | 2 | 1 | 3 |
| *Gymnodiptychus pachycheilus* | Xinghai, Qinghai | Yellow River (7) | Yellow River | JQ082349* | 0 | 2 | 0 | 2 | 1 | 0 |
| *Gymnodiptychus dybowskii* | Heshuo, Xinjiang | Longka River (10) | Tarim River | JQ082350* | 1 | 2 | 2 | 2 | 1 | 0 |
| *Gymnocypris eckloni* | Madoi, Qinghai | Zhalin Lake (14) | Yellow River | JQ082351* | 0 | 1 | 0 | 2 | 2 | 4 |
| *Gymnocypris przewalskii* | Gonghe, Qinghai | Qinghai Lake (12) | Qinghai Lake | JQ082353* | 0 | 1 | 0 | 2 | 2 | 4 |
| *Gymnocypris scolistomus* | Jiuzhi, Qinghai | Sunmcuo Lake (13) | Yellow River | JQ082352* | 0 | 2 | 0 | 2 | 2 | 4 |
| *Oxygymnocypris stewartii* | Lhasa, Tibet | Lhasa River (1) | Yarlungzangbo River | JQ082354* | 0 | 2 | 0 | 2 | 2 | 4 |
| *Schizopygopsis stoliczkai* | Ali, Tibet | Sênggê Zangbo River (11) | Indus River | JQ082365* | 2 | 0 | 0 | 2 | 1 | 2 |
| *Schizopygopsis pylzovi* | Datong, Qinghai | Heiquan River (8) | Huangshui River | JQ082355* | 2 | 1 | 0 | 2 | 1 | 2 |
| *Schizopygopsis malacanthus* | Chengdoi, Qinghai | Yarlun River (4) | Yangtze River | JQ082357* | 2 | 1 | 0 | 2 | 1 | 2 |
| *Schizopygopsis anteroventris* | Zadoi, Qinghai | Jiqu River (2) | Lancang River | JQ082358* | 2 | 1 | 0 | 2 | 1 | 2 |
| *Schizopygopsis younghusbandi* | Lhasa, Tibet | Lhasa River (1) | Yarlungzangbo River | JQ082361* | 2 | 1 | 0 | 2 | 1 | 2 |
| *Schizopygopsis chengi* | Banma, Qinghai | Dadu River (5) | Yangtze River | JQ082360* | 2 | 1` | 0 | 2 | 1 | 2 |
| *Schizopygopsis kessleri* | Germu, Qinghai | Germu River (9) | Qiadam Basin | JQ082356* | 2 | 1 | 0 | 2 | 1 | 2 |
| *Schizopygopsis kialingensis* | Langmusi, Sichuan | Kialing River (6) | Yangtze River | JQ082359* | 1 | 1 | 0 | 2 | 1 | 1 |
| *Chuanchia labiosa* | Madoi, Qinghai | Zhalin Lake (14) | Yellow River | JQ082362* | 1 | 1 | 3 | 2 | 1 | 1 |
| *Platypharodon extremus* | Xinghai, Qinghai | Yellow River (7) | Yellow River | JQ082363* | 2 | 0 | 2 | 2 | 1 | 2 |
| *Herensteinia microcephalus* | Germu , Qinghai | Jinsha River (3) | Yangtze River | JQ082364* | 2 | 1 | 2 | 3 | 1 | 2 |

* This study.

Morphological characters and character coding used in this study: LJM, lower jaw morphology (0 = no horny sheath, 1 = with blunt outer horny sheath or inner horny membrane, 2 = with sharp outer horny sheath); PB, shape of the pharyngeal bones (0 = broad, 1 = intermediate, 2 = narrow); PTS, shape of the pharyngeal teeth (0 = spoon-shaped teeth, 1 = molariform teeth, 2 = spatulated teeth, 3 =conical teeth); PTR, pharyngeal teeth rows (0 = three, 1 = four, 2 = two, 3 = one); MP, mouth position (0 = subinferior, 1 = inferior, 2 = terminal); Diet (0 = benthic aquatic insects, few algae and aquatic plants, 1 = benthic invertebrates, few aquatic insects and periphytic algae, 2 = periphytic algae and some organic debris, 3 = periphytic algae and a few benthic invertebrates, 4 = plankton, a few algae and aquatic plants, 5= juvenile freshwater fishes and a few benthic aquatic insects.
